# Early Wearing-Off Effect of OnabotulinumtoxinA in Chronic Migraine: A Prospective Real-Life Study

**DOI:** 10.3390/jcm12165360

**Published:** 2023-08-17

**Authors:** Joana Rodríguez-Montolio, María Pilar Navarro-Pérez, Monserrath Almeida-Zurita, Sonia Santos-Lasaosa

**Affiliations:** 1 Neurology Department, Hospital Clínico Universitario Lozano Blesa, 50009 Zaragoza, Spainssantos@salud.aragon.es (S.S.-L.); 2 Aragon Institute for Health Research (IIS Aragón), 50009 Zaragoza, Spain

**Keywords:** migraine, chronic migraine, treatment, Onabotulinumtoxin A, wearing-off, clinical predictor

## Abstract

Objective: Chronic migraine (CM) is a significant public health problem that affects 2.2% of the global population. Onabotulinumtoxin A (OnabotA) is a safe and effective prophylactic treatment for patients with CM. The standard injection interval for OnabotA is 12 weeks. Nevertheless, some patients experience a wearing-off effect (WOE) in the weeks preceding the next scheduled cycle. The objectives of this study are to determine the prevalence of early WOE, to analyze variables that could be clinical predictors and to specify which interval is the most appropriate to define the existence of this phenomenon. Methods: This is a prospective single-center study of consecutive adult patients with CM who, after failing previous prophylactic therapies, started OnabotA treatment following the PREEMPT protocol between June and December of 2021. Results: A total of 59 patients (93.2% female, age 44 ± 12 years) were included. A total of 37 patients (64.9%) fulfilled medication overuse criteria. Of the total patients, 40.6% reported WOE and this was more frequent after the first cycle (35.6%). Depression and anxiety disorder was a statistically significant clinical predictor of WOE (OR 3.4; CI 95% 1.22–10.84; *p* = 0.028). A better cut-off point to consider WOE seems to be at 10 weeks. Conclusions: Early WOE is common in patients on OnabotA treatment for CM. Individualizing the standard 12-week injection, using total doses of 195 U, and managing psychiatric comorbidities with pharmacological and non-pharmacological strategies may improve treatment outcomes and reduce OnabotA WOE.

## 1. Background

Migraine is a common disabling primary headache disorder with a significant and debilitating impact on physical, social, and occupational functioning [1,2]. It is considered the second leading cause of disability worldwide and the leading cause among young women [3,4]. However, migraine is also an under-diagnosed and under-treated disease that needs prevention and a multidisciplinary approach [2].

Chronic migraine (CM), which usually appears as a result of the worsening pain frequency in Episodic Migraine (EM), is defined as a headache that occurs on 15 or more days of the month for more than 3 months, of which the headache has the phenotypic characteristics of a migraine on at least 8 days of the month [5].

CM is a major public health problem affecting 2.2% of the world’s population. It significantly reduces health-related quality of life and decreases productivity [6]. Headache-related resource utilization, medication use, productivity loss, and total costs are significantly higher in CM than in EM [7], especially in those patients with resistant and refractory migraine [8].

Psychiatric comorbidities, particularly mood and anxiety disorders, are more common among patients with CM than among those with EM. It is important to identify anxiety and depressive symptoms and to treat them appropriately because comorbid psychiatric conditions increase migraine-related disability and impact, decrease health-related quality of life, and influence treatment response with poor therapeutic outcomes [9,10,11].

CM is often associated with medication overuse that may aggravate the headache itself and cause Medication Overuse Headache (MOH). MOH is a secondary headache caused by the overuse of analgesics or others medications such as triptans to abort acute migraine attacks that usually ameliorates following the interruption of regular medication use [12].

Preventive therapies for CM are used to reduce the headaches’ frequency, intensity, and duration and to indirectly reduce the consumption of healthcare resources [6,13]. Antiepileptic drugs (e.g., topiramate, valproate), antihypertensive agents (e.g., beta blockers, calcium channel blockers, aldosterone receptor blockers), and tricyclic antidepressants (e.g., amitriptyline, nortriptyline) have been used in the prevention of CM [11]. However, adherence to oral preventive medication often decreases over time, in part due to undesirable side effects such as drowsiness, decreased attention, dizziness, fatigue, hypotension, and weight gain [11,14].

Onabotulinumtoxin A (OnabotA) is a neuromuscular blocking agent produced by the bacterium *Clostridium botulinum*. It was approved for the preventive treatment of CM by the Food Drug Administration (FDA) in 2010 based on the results of the Phase III Research Evaluating Migraine Prophylaxis Therapy (PREEMPT) [15,16,17]. The mechanism of action of OnabotA has not yet been fully elucidated, but it has been postulated that it prevents migraine attacks by inhibiting peripheral sensitization and thus indirectly reducing the progression of central sensitization. It seems to block the peripheral release of inflammatory neuropeptides such as Substance P and calcitonin gene-related peptide (CGRP). In addition, it also blocks the translocation of membrane receptors to the surface of sensory neurons, such as the vanilloid transient receptor potential channel (TRPV1) [14,18,19].

OnabotA has been used in Spain since 2012 [20] and is an effective, safe, and tolerable long-term prophylactic treatment for CM [21,22,23] that improves multiple measures of headache symptoms and reduces headache-related disability [17,24]. The administration of OnabotA used in the PREEMPT protocol requires the intramuscular injection of 155 U of OnabotA at 31 sites in 7 muscles of the head and neck. An additional up to 40 U of OnabotA can be injected into 8 additional sites across 3 muscles of the head and neck using the “follow the pain” strategy [21].

The established interval between cycles of OnabotA is 12 weeks, although depending on the response, this can be extended to 24 weeks in some patients. A series of retrospective studies have been published in recent years to analyze the existence of the wearing-off effect (WOE) [6,14,19,25,26]. WOE is a reduction in the therapeutic benefit of OnabotA near the end of the treatment cycle [19]. It is a clinical worsening 8-10 weeks after an initial good response [6]. There is no agreement in the scientific community regarding the establishment of which week we should consider that the patient has a WOE rather than a lack of response to treatment. Most authors consider week 8 as the cut-off point [14], although some studies delay it to week 10 [19].

The main objective of this study is to determine the prevalence of the WOE in patients with CM treated with OnabotA in the first two cycles. The secondary objectives are to analyze the demographic and clinical variables that could be clinical predictors of WOE and to specify which week seems more appropriate to define the cut-off point for WOE: week 8 or week 10.

## 2. Material and Methods

This is an observational analytical study with a prospective cohort design including patients with CM who start prophylactic treatment with OnabotA in the Headache Unit of a tertiary hospital.

Eligible patients were those fulfilling the International Classification of Headache Disorders III (ICHD-III) criteria [5] for CM who started sessions of OnabotA pericraneal injections following the PREEMPT protocol between June and December of 2021. According to the current guidelines of the Headache Study Group of the Spanish Society of Neurology [27], OnabotA was initiated in patients who showed insufficient response, absence of tolerability, or contraindications to at least two oral migraine prophylactic treatments, taking into account that topiramate should be considered as a preventive oral treatment in CM (Grade: strong recommendation; high-quality evidence).

All patients completed a minimum of 2 cycles of OnabotA treatment. Patients were allowed to continue with their preventive oral medication during treatment with OnabotA with no dose increase. No new preventive oral medication was allowed to be started.

An amount of 195 U was used as the first dose in patients with long-term CM, with medication overuse and/or other causes of chronic pain. An amount of 155 U was used as the first dose in patients without comorbidities.

Exclusion criteria were (1) patients aged <18 years old, (2) patients without confirmed consent, and (3) patients with no possible follow up. Patients who fulfilled criteria for medication overuse and patients with comorbidities such as anxiety, depression or fibromyalgia were not excluded.

Data collected from patients included age, sex, comorbidities, age at migraine onset, years with CM, and preventive oral medication. They were also asked to keep a conventional headache diary during OnabotA treatment that included headache and migraine days and analgesic and triptan intake days. Migraine-related disability was measured using Migraine Disability Assessment (MIDAS) and Headache Impact Test-6 (HIT-6). Headache intensity was measured based on Numerical Rating Scale (NRS). In order to search for possible comorbid anxiety or depression, patients carried out the Hospital Anxiety and Depression Scale (HADS). This is a 14-item self-assessment questionnaire used to screen for symptoms of anxiety and depression. It consists of a depression subscale (HADS-D, 7 items) and an anxiety subscale (HADS-A, 7 items). The total score on each subscale ranges from 0 to 21 points, with scores > 8 indicating clinically relevant depression or anxiety [28].

Data were compared between the baseline visit and visits at 8, 10, and 12 weeks after each treatment session.

Response to OnabotA was defined as a reduction in headache/migraine days of at least 50% after treatment cycle. Wearing off was defined as a reduction in headache/migraine days at week 8 or week 10 but not at week 12.

Safety and tolerability were assessed by reviewing the frequency of adverse events (AE). AE were determined at the corresponding visits using patient self-report and general questions.

Statistical analysis was performed using the Statistical Package for the Social Sciences (SPSS; version 23.0). The data were summarized using descriptive statistics. Discrete variables were expressed as the number of cases and their percentage.

The Kolmogorov–Smirnov test was used to ensure the normality of the data. Continuous variables were expressed as mean and standard deviation (SD). Categorial variables were expressed as percentages and frequencies. Student’s *t* test and Wilcoxon test were performed to compare means. Chi square test was used to compare categorical variables. *p* ≤ 0.05 was considered statistically significant.

The study was conducted in accordance with the declaration of Helsinki and is reported in accordance with the STrengthening the Reporting of OBservational studies in Epidemiology (STROBE) statement [29]. It was approved by the Local Ethics Committee of Clinical Research, based at the Aragon Institute for Health Research (IIS Aragon). Patient anonymity and compliance with the Data Protection Laws were maintained at all times.

## 3. Results

A total of 59 patients were included, 55 females (93.2%) and 4 males. The mean age at first treatment cycle was 44 ± 12 years. The mean time with CM was 5.09 ± 4.4 years and patients had previously received 3.21 ± 1.2 oral preventive treatments. Amitriptyline (84.7%), flunarizine (72.9%), and topiramate (62.7%) were the most frequently used preventive medications. Migraine with aura criteria were met by 13.6% of the patients.

At baseline visit, the mean headache days per month was 23.07 days and the mean migraine days per month was 16.34 days. The mean analgesics intake days per month was 15.71 and the mean triptan intake days was 8.96. A total of 37 patients (64.9%) fulfilled the medication overuse criteria. The main pain intensity was 8.24. Most patients had a severe (>60) HIT-6 score. Additional data are shown in Table 1.

Follow the pain protocol with additional OnabotA injections until 195 U was used in 50 patients (84.7%) in the first cycle and in all of them in the second one. 

After the first treatment cycle, 18 patients (30.5%) remitted to EM. In 32 patients (52.5%), the migraine/headache days per month improved by at least 30%. A total of 27 patients (45.8%) fulfilled the medication overuse criteria. The main pain intensity was reduced to 7.3.

No adverse effects were reported.

WOE was observed in 24 patients (40.6%) and was more frequent after the first cycle (35.6%). A total of 14 patients (23.7%) reported WOE at 10 weeks after the first cycle. After the second cycle, 7 patients (11.9%) reported WOE at both the 8 and 10 week visits (Table 2).

The demographic and clinical data are compared in patients with and without WOE in Table 3. Anxiety and depression and anxiety disorder were associated with WOE (*p* = 0.05 and 0.018, respectively). A logistic regression model was performed using WOE as the dependent variable. Depression and anxiety disorder reached statistical significance with an OR of 3.4 (CI 95% 1.22–10.84, *p* = 0.028). No other statistically significant difference was seen between any clinical or demographical variable and WOE, nor OnabotA units in the first cycle.

Of these 24 patients, 13 reported 1 WOE event and 11/24 patients reported 2 WOE events. In Table 4, these patients are compared. The MIDAS score was higher in patients with 1 WOE (53.65% vs. 12.95%; *p* = 0.005). The years of CM were also higher in patients with 1 WOE (*p* = 0.009).

## 4. Discussion

The objectives of this study were to determine the prevalence of the WOE in patients with CM treated with the first two cycles of OnabotA, to analyze the demographic and clinical variables that could be clinical predictors of WOE, and to specify which week is the best cut-off point for defining the existence of WOE.

OnabotA is an effective prophylactic treatment in CM that has the potential to suppress central sensitization by blocking the peripheral release of neurotransmitters and inhibiting those peripheral signals to the central nervous system [19,30]. The WOE may indicate a disruption in the ability of OnabotA to break this inflammatory loop that involves the nociceptive neurons that promote peripheral and central sensitization [19].

In our sample, in order to approach real clinical practice, patients with psychiatric comorbidities and with medication overuse were also included. Our study population included predominantly female migraineurs in their 40s with medication overuse and some years of CM evolution.

OnabotA treatment resulted in improvements in migraine and headache days, triptan and analgesic intake days, and headache intensity, as well as MIDAS and HIT-6 scores after just the first cycle. In total, 30.5% of patients fulfilled the criteria for EM 12 weeks after the first treatment session. In addition, no adverse effects were reported. In agreement with previous real-life studies, our data also confirmed that OnabotA is a safe and rapidly effective prophylactic treatment in CM.

To our knowledge, this is the first prospective study to analyze the WOE in the first cycles of OnabotA treatment. Our results suggest that almost 40% of patients with CM receiving OnabotA treatment experience WOE during the last weeks prior to the scheduled reinjection. Previous studies have described a variable prevalence of WOE that ranges from 23.3% [6] to 63% [14].

The physiological basis for this phenomenon is unclear and may represent the effects of external factors such as variation in disease progression, lifestyle, and CM management [19]. Stressful life events may affect the subjective experience of WOE. Natural fluctuations in migraine characteristics over time may also play a role in the perception of WOE, as well as the different mechanisms of OnabotA on nociception, including its alteration of muscle tone, anti-inflammatory activity, and impact on peripheral and central afferences [14].

The duration of the specific antinociceptive properties of OnabotA has not been investigated in humans. The heterogeneity in the duration among individual patients could be related to variable rates of neuronal regenerative capacity and/or natural resistance to the effects of the toxin [19].

In our study, 35.6% of patients reported WOE after the first cycle of OnabotA and 23.8% reported it after the second one. Therefore, WOE was more frequent after the first OnabotA infiltration. This could be explained by the cumulative benefit of OnabotA over time, as demonstrated in the PREEMPT clinical studies [15,16,17]. Repeated injections over time are able to increase the benefit obtained after the first cycle of treatment [11].

The early wearing off response has been described as unpredictable [6]. Masters et al. [14] did not find demographic or baseline characteristics associated with WOE. Khan et al. [19] described significant differences in motion sickness and infectious meningitis when comparing patients with and without WOE, and in opioid use and anxiety disorder when comparing patients with 1 or >2 WOE events. Quintas et al. [6] identified that there was a trend for patients with more frequent headaches at baseline experiencing more WOE events. In our sample, years of CM and migraine-related disability were associated with earlier WOE events and differences between patients with 1 vs. 2 WOE events, reaching statistical significance.

Anxiety with and without concomitant depressive disorder was found to be associated with WOE. Migraine is a highly prevalent neurological disorder which is commonly linked with psychiatric comorbidities. Anxiety and depression, that are common among patients with CM impacting on migraine-related disability and quality of life, seem to be clinical predictors of OnabotA WOE. This is important in clinical practice because detecting and treating these symptoms by pharmacological and non-pharmacological treatments such as psychotherapies with a cognitive behavioral approach may be useful in therapeutic strategies for migraine prevention [31]. This multidisciplinary management can influence the clinical course of migraine, treatment response, and clinical outcome and may reduce WOE in a subgroup of patients. This finding should not be considered an anxiety-anticipatory phenomenon because patients were not informed at baseline about the existence of WOE.

There is no agreement regarding on which week the WOE phenomenon should be considered. More than half of the patients included in our study reported WOE at week 10. A total of 14 of the 21 patients (66.67%) who experienced WOE after the first cycle, and 7 of 14 patients (50%) after the second one, reported worsening headache at 10 weeks. Based on these data, it seems that week 10 is a better cut-off point than week 8.

The standard injection interval of 12 weeks should be individualized for each patient, especially in those patients with WOE, although shortening this interval time between cycles is not yet recommended in OnabotA clinical guidelines in CM.

Although we did not find statistically significantly differences when comparing OnabotA units in the first cycle, the infiltration of an additional 40 units should also be considered in patients with WOE from the first cycle. Both OnabotA 155 U and 195 U significantly reduce the number of headache and migraine days and acute pain medication intake days. However, OnabotA 195 U proved to be superior to 155 U in all efficacy measures with the same safety and tolerability [11]. In our sample, most patients were started with 195 U because they usually suffer MOH or other chronic pain.

The incidence of treatment-emergent EA, such as neck pain, eyelid ptosis, and musculoskeletal stiffness, is higher in the first cycles because it typically decreases with repeated treatment with OnabotA [32]. Nevertheless, although we analyzed the first treatment cycles, our patients did not report EA.

Our study had some limitations. This is a single center study, with a small sample size with no results regarding long-term OnabotA WOE. Our data are limited to patients who received their first two cycles of OnabotA and the result cannot be generalized to later cycles because we focused on early OnabotA WOE. Nevertheless, we would like to highlight the prospective design of the study, the analysis of multiple variables as a possible clinical predictor of WOE, and the accuracy of the data used through headache diaries.

Future prospective studies focusing on psychiatric comorbidities are needed in order to analyze whether a better multidisciplinary and individualized management reduces OnabotA WOE in some patients.

## 5. Conclusions

WOE is common in patients with CM receiving OnabotA and it is defined as a worsening of headache/migraine days, after a good response, in the last 2–4 weeks between treatment cycles. We have described anxiety and anxiety-depressive disorder as clinical predictors of WOE. A better cut-off point seems to be at 10 weeks. Individualizing the standard 12-week injection and using total doses of 195 U may reduce OnabotA WOE. An interdisciplinary management of migraine and psychiatric comorbidities with pharmacologic and non-pharmacologic strategies is essential in order to improve treatment outcomes and to reduce migraine disability and OnabotA WOE.

## Figures and Tables

**Table 1:** Demographic and headache characteristics in patients in baseline and visits 2 and 3 (N = 59).

	Baseline	Visit 2 (after 1st Cycle)	Visit 3 (tras 2nd Cycle)
Female sex n (%)	55 (93.2)		
Age mean (SD)	44 (12)		
Years of CM mean (SD)	5.09 (4.4)		
Migraine with aura n (%)	8 (13.6)		
Obesity n (%)	7 (11.9)		
Sleep disorders n (%)	14 (23.7)		
Depression n (%)	21 (35.6)		
Anxiety n (%)	21 (35.6)		
Depression and anxiety disorder n (%)	26 (44.1)		
Arterial hypertension n (%)	4 (6.8)		
Fybromyalgia n (%)	4 (6.8)		
Medication overuse n (%)	37 (64.9)	27 (45.8)	25 (42.4)
Previous OPT mean (SD)	3.21 (1.28)		
Topiramate n (%)	37 (62.7)		
Beta-blockers n (%)	32 (54.2)		
Flunarizine n (%)	43 (72.9)		
Anti Hypertensive n (%)	2 (3.4)		
Amitriptyline n (%)	50 (84.7)		
Others n (%)	12 (20.3)		
Migraine days mean (SD)	16.34 (8.26)	10.92 (6.95)	11.19 (7.34)
Headache days mean (SD)	23.07 (7.38)	15.81 (9.53)	15.24 (9.46)
Triptan intake days mean (SD)	8.96 (9.66)	5.91 (6.1)	5.95 (6.23)
Analgesic intake days mean (SD)	15.71 (12.06)	10.93 (10.74)	10.49 (10.68)
Intensity of headache mean (SD)	8.24 (1.8)	7.3 (1.56)	7.28 (1.50)
HIT-6 mean (SD)	67.11 (6.97)	63.85 (6.07)	63.49 (7.34)
MIDAS mean (SD)	71.39 (65.73)	44 (39.96)	46.02 (43.99)
Conversion to EM n (%)		18 (30.5)	16 (27.1)

SD = Standard deviation; CM = Chronic migraine; OPT = Oral preventive treatment; EM= Episodic migraine; HIT-6 = Headache Impact Test-6; MIDAS = Migraine Disability Assessment Scale.

**Table 2:** Patients with Wearing Off Effect (WOE) (n = 24).

WOE in 24 Patients (40.6%)				
	Cycle 1		Cycle 2	
	WOE 8-Week	WOE 10-Week	WOE 8-Week	WOE 10-Week
Patients	7 (11.9%)	14 (23.7%)	7 (11.9%)	7 (11.9%)
Total	21 (35.6%)		14 (23.8%)	

Percentages are calculated based on total of patients.

**Table 3:** Comparisons of clinical data of patients with and without WOE.

	Without WOE (n = 35)	With WOE (n = 24)	*p* Value
Female sex n (%)	31 (88.6)	24 (100)	0.115
Age mean (SD)	45.57 (11.6)	41.88 (12.55)	0.25
Years of CM mean (SD)	5.75 (5.25)	4 (2.22)	0.162
Migraine with aura n (%)	6 (17.14)	2 (16.67)	0.33
Depression n (%)	10 (28.57)	11 (45.83)	0.174
Anxiety n (%)	9 (25.71)	12 (50)	**0.05**
Depression and anxiety disorder n (%)	11 (31.4)	15 (62.5)	**0.018**
Medication overuse n (%)	24 (68.57)	13 (54.17)	0.327
Previous OPT mean (SD)	3.23 (1.42)	3.17 (1.07)	0.875
Migraine days mean (SD)	14.94 (7.68)	18.33 (8.8)	0.125
Headache days mean (SD)	23.29 (7.37)	22.75 (7.54)	0.787
Triptan intake days mean (SD)	8.91 (9.07)	9.05 (10.84)	0.96
Analgesic intake days mean (SD)	15.35	16.39 (13.2)	0.77
Intensity of headache mean (SD)	8.09 (2.1)	8.5 (1.15)	0.422
HIT-6 mean (SD)	67.1 (7.49)	67.14 (6.39)	0.98
MIDAS mean (SD)	83.18 (72.64)	52.86 (49.17)	0.09
155 U OnabotA 1st cycle n (%)	3 (8.57)	6 (25)	0.18

WOE = Wearing Off Effect; SD = Standard deviation; CM = Chronic migraine; OPT = Oral preventive treatment; EM = Episodic migraine; HIT-6 = Headache Impact Test-6; MIDAS = Migraine Disability Assessment Scale.

**Table 4:** Comparisons of clinical data of patients reporting one and two WOE.

	WOE in 1 Cycle (n = 13)	WOE in 2 Cycles (n = 11)	*p* Value
Years of CM mean (SD)	4.91 (2.46)	2.62 (0.51)	**0.009**
Migraine days mean (SD)	20.85 (9.44)	15.36 (7.29)	0.131
Headache days mean (SD)	24.23 (7.94)	21 (6.97)	0.306
Triptan intake days mean (SD)	11.18 (12.63)	6.44 (8.11)	0.345
Analgesic intake days mean (SD)	18.3 (12.72)	14 (14.26)	0.5
Intensity of headache mean (SD)	8.5 (1.38)	8.7 (0.75)	1
HIT-6 mean (SD)	67.75 (7.31)	66.4 (8.11)	0.634
MIDAS mean (SD)	80.82 (53.65)	22.1 (12.95)	**0.005**

CM = Chronic migraine; SD = Standard deviation; HIT-6 = Headache Impact Test-6; MIDAS = Migraine Disability Assessment Scale.

## Data Availability

Not applicable.
